# ACOX1 gain-of-function post-mortem neuropathology is distinct from ACOX1 loss-of-function: case report and literature review

**DOI:** 10.17879/freeneuropathology-2025-8894

**Published:** 2025-10-10

**Authors:** Zita Hubler, Kaleigh Filisa Roberts, Nima Sharifai, Julia Sim, Sophia A. Hung, Grace E. Robvais, Alan Pestronk, Robert E. Schmidt, Sonika Dahiya, Robert C. Bucelli

**Affiliations:** 1 Washington University in St. Louis, Department of Pathology and Immunology, USA; 2 University of Maryland, School of Medicine, Department of Pathology, USA; 3 Washington University in St. Louis, Department of Neurology, USA; 4 Washington University in St. Louis, Department of Biology, USA

**Keywords:** Mitchell Syndrome, ACOX1, Pseudo-neonatal adrenoleukodystrophy, Case report

## Introduction

Mitchell Syndrome is a newly described progressive childhood neurodegenerative disorder characterized by myeloneuropathy, hearing loss, and skin changes, progressing to encephalopathy, paralysis, and death before the third decade [1]. Mitchell Syndrome is caused by a gain-of-function (GOF) missense variant in peroxisomal Acyl-CoA oxidase 1 (ACOX1) [1]. ACOX1 is the rate-limiting enzyme of peroxisomal β-oxidation, including very long-chain fatty acids (VLCFAs). ACOX1 loss-of-function (LOF), known as pseudo-neonatal adrenoleukodystrophy, is associated with a spectrum of genetic variants, the accumulation of VLCFAs in plasma, hypotonia, seizure, early-onset leukodystrophy, and death in early childhood [2]. Both gain- and loss-of-function results in myelin loss based on imaging and animal studies [1,3]. We report, for the first time, the postmortem neuropathologic findings in Mitchell Syndrome (ACOX1 GOF), compare them to ACOX1 LOF, and discuss the implications for future studies.

## Case presentation

**Figure 1: Perimortem imaging and gross pathology.**
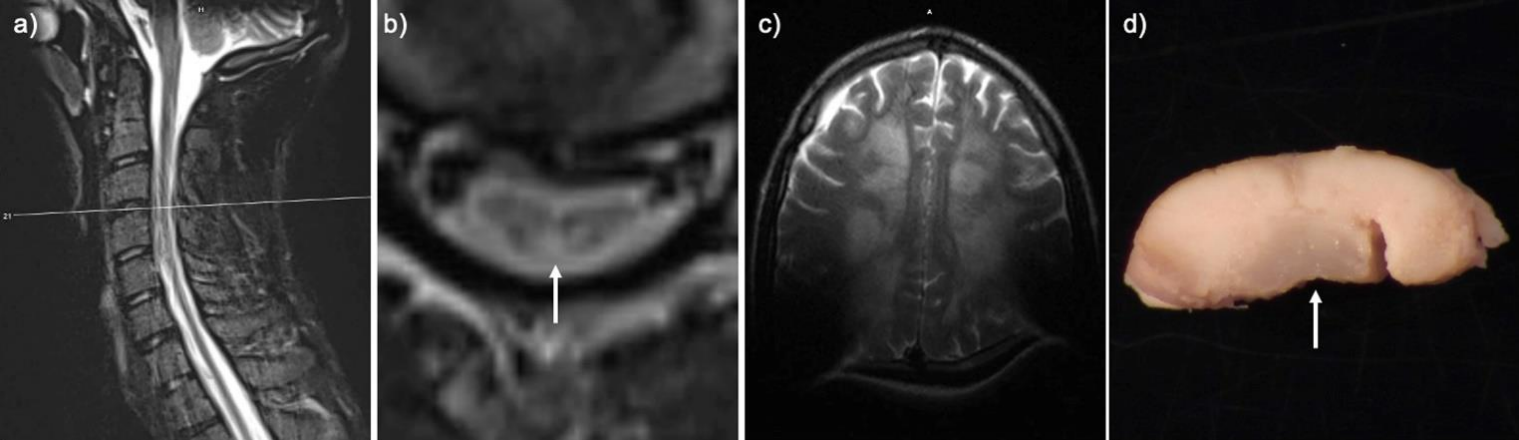
T2-weighted Turbo Spin Echo (TSE) sequence in the transverse (TRA) plane of **a**) sagittal cervical cord, **b**) transverse cervical cord showing hyperintensity of the dorsal column (arrow), and **c**) axial brain with artifact due to cochlear implants. **d**) Post fixation cross-section of the cervical cord with translucency of the dorsal column (arrow).

An 11-year-old boy presented with ocular keratopathy, clumsiness, sensory ataxia, bilateral hearing loss, and hyporeflexia. He developed progressive myeloneuropathy; imaging showed a longitudinally extensive lesion preferentially involving the dorsal columns (**Fig. 1a–b**). The patient was extensively investigated, and other known causes of longitudinally extensive transverse myelopathy were ruled out, including neoplastic/paraneoplastic, infectious, mitochondrial and other known metabolic disorders, neurodegenerative, toxic exposures, and autoimmune conditions. For additional details on clinical course please see paper Chung et al., 2020 supplemental document Methods S1 [1]. Initially, the brain was relatively unaffected on imaging. The spinal cord disease progressed to involve the anterolateral cord. At age 13 a peripheral nerve biopsy showed mildly abnormal myelin profiles and ongoing and chronic axon loss [1]. An Undiagnosed Diseases Network evaluation uncovered a novel de novo heterozygous ACOX1 N237S variant. He suffered a relapsing and remitting course with overall progressive decline despite treatment with N-acetyl cysteine, plasmapheresis, intravenous immunoglobulin, corticosteroids, rituximab, cyclophosphamide, and tocilizumab [1]. At 19, he was admitted for a urinary tract infection and developed encephalopathy and neuromuscular respiratory failure requiring mechanical ventilation. Brain MRI showed new, diffuse, bilateral subcortical Fluid-Attenuated Inversion Recovery (FLAIR) and T2-weighted Turbo Spin Echo (TSE) hyperintensities with U-fiber sparing, consistent with disease progression (**Fig. 1c**). The patient passed away after elective extubation and underwent a complete autopsy within 24 hours after death. To date, an additional 30 cases have been identified.

## Pathology

The weight of the unfixed brain was 1510 grams (reference range 1250–1400 grams), possibly increased due to edema. The intact brain was grossly unremarkable. Microscopic sections of the cortex revealed variable scattered white matter pallor and focally increased rarefaction. The cingulate gyrus showed white matter pallor with sparing of the U-fibers, confirmed by Luxol Fast Blue and Periodic Acid-Schiff (LFB-PAS) stain. A neurofilament immunostain showed these areas of white matter to have a disrupted axonal array with numerous axonal swellings. Mildly increased numbers of oligodendrocytes were noted in the demyelinated and adjacent areas. A CD68 immunostain of the cingulate gyrus highlighted increased microglia and perivascular macrophages in the white matter. The occipital lobe showed modest white matter pallor, without neuronal dropout, while the superior frontal lobe shows no obvious white matter pathology. The cortical gray matter in the affected lobes showed a normal number of neurons and intact laminar architecture. Sections of the basal ganglia showed relative neuronal preservation of the caudate and putamen without significant astrocytosis. Sections of the thalamus showed a normal neuronal density and glial component. The hippocampus showed neuronal dropout in the CA4 region, with Hirano bodies in CA1 and the subiculum. The midbrain and pons were unremarkable. The medulla showed white matter pallor within the medullary pyramids, mild neuronal dropout, and associated gliosis in the inferior olivary nucleus. The cerebellum was unremarkable with a well-preserved complement of cortical Purkinje, granule, and dentate nucleus neurons.

**Figure 2: Spinal cord histopathology.**
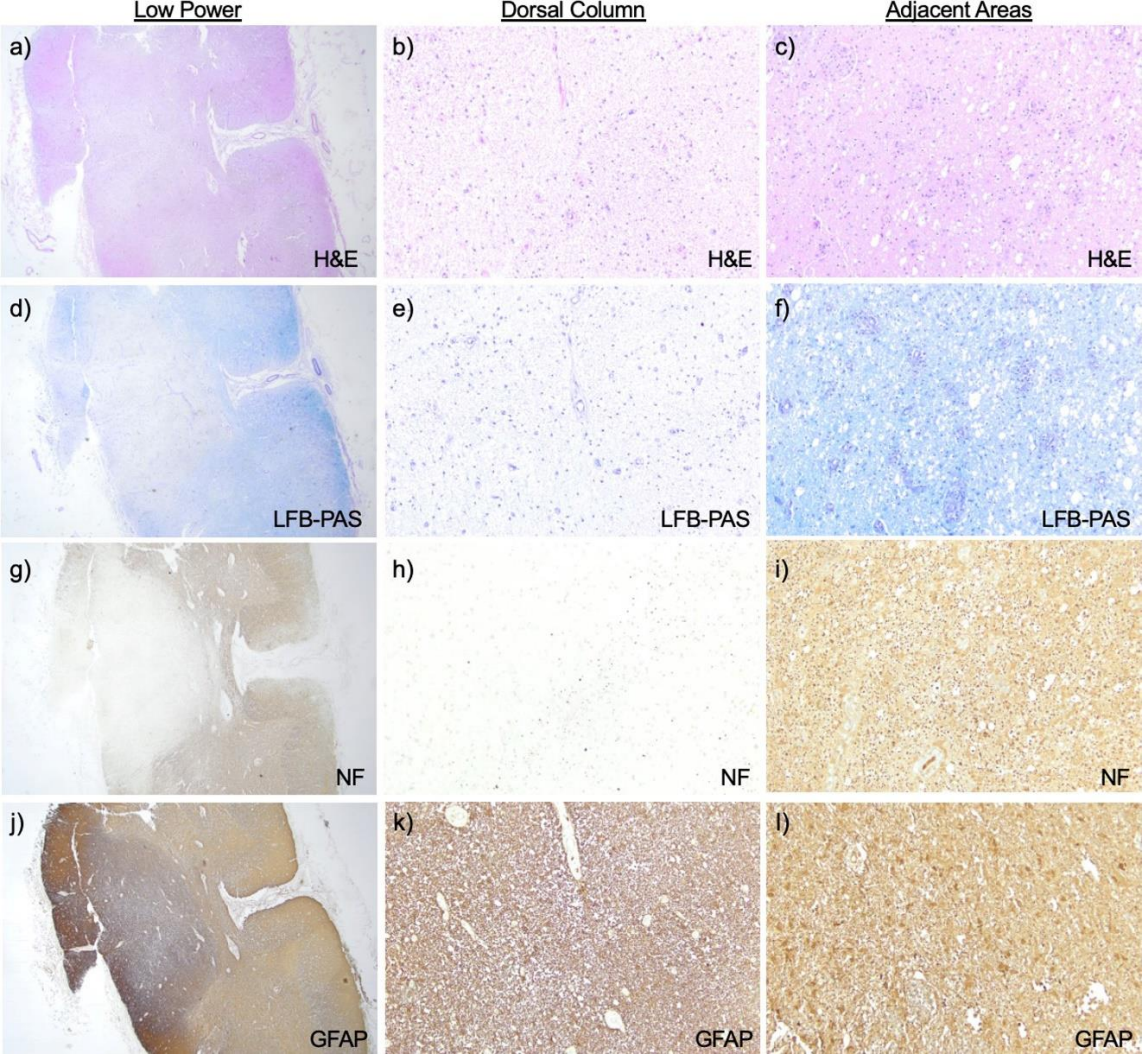
H&E at **a**) low power cross sections (1.25x) of cervical spinal cord and **b**) high power section of the dorsal column shows destruction, while **c**) adjacent areas show relative preservation. Loss of myelin is seen on LFB-PAS **d–f**), loss of axons by neurofilament **g–i**), and reactive gliosis by Glial Fibrillary Acidic Protein (GFAP) **j–l**) in the dorsal column. **Clicking into the picture will lead you to the full virtual slide** https://doi.org/10.57860/min_dts_000024

**Figure 3: Macrophage staining, dorsal root ganglion and peripheral nerve pathology.**
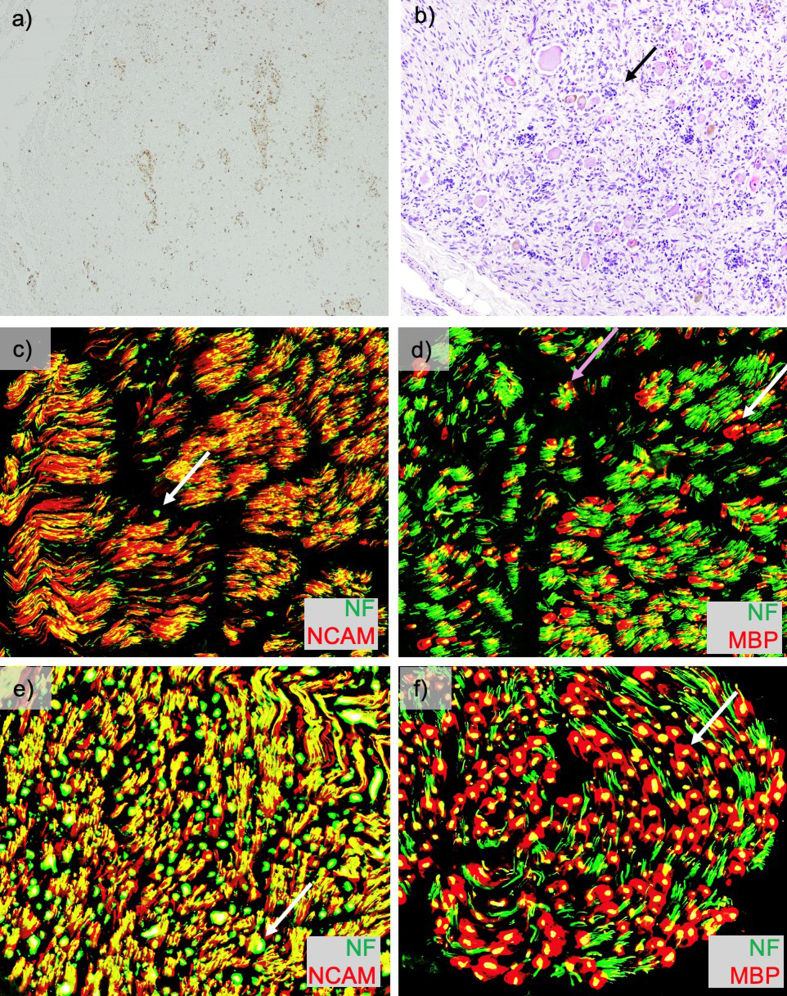
Macrophage staining, dorsal root ganglion and peripheral nerve pathology. **a**) CD68 stain of spinal cord (4x) showing active macrophage infiltration. **b**) Dorsal root ganglia (10x) showing frequent Nageotte nodules (arrow). Frozen sections of sciatic nerve were immunostained for neural cell adhesion molecule (NCAM), neurofilament (NF), and myelin basic protein (MBP). Overlay images of **b**) NCAM (red)/NF (green) (200x) show a severe loss of large greater than small, myelinated axons (very few large green dots, example white arrow) and a moderate loss of small, non-myelinated axons (red staining relative to yellow staining). Overlay images of **c**) MBP (red)/NF (green) (200x) again show the loss of large (white arrow) greater than small (purple arrow), myelinated axons, empty MBP sheaths (evidence of ongoing axon loss) and small axons with associated MBP staining (immature/regenerating axons). For additional details on the pathologic correlates of this pattern of staining findings, see Pestronk et al., 2023 [4]. For a comparison to these stains on the patient’s sural nerve from six years prior see Chung et al., 2020 supplemental figure 6 [1]. For comparison, control frozen sural nerve at 200x stained with **e**) overlay images of NCAM (red)/NF (green) showing green dots with white arrow, and** f**) MBP (red)/NF (green) showing a large myelinated fiber with white arrow. (used with permission from https://neuromuscular.wustl.edu/pathol/nervenl.htm#axsur)

Grossly, the spinal cord showed translucency and discoloration of the dorsal column throughout, but particularly in the cervical cord (**Fig. 1d**). Microscopically there was total destruction of the dorsal columns with loss of myelin, loss of axons, and reactive gliosis (**Fig. 2a–b, d–e, g–h, j–k**). Areas near the dorsal column showed relative preservation of the axonal array with gliosis (**Fig. 2c, f, i, l**) and active macrophage infiltrate showed with a CD68 stain (**Fig. 3a**). In addition, there was pallor and vacuolation in the anterior and lateral corticospinal tracts. CD68 immunostain highlighted macrophages most prominent in the corticospinal tract in a perivascular distribution with PAS-positive intracytoplasmic material corresponding to digested myelin. There were few scattered oligodendrocyte transcription factor 2 (Olig2) positive oligodendrocytes. The cervical and thoracic cord showed neuron loss and atrophy with associated gliosis, greater in the dorsal horn than the ventral horn. Sections of the lumbosacral cord showed similar pathology that was less severe. Frozen sections of sciatic nerve were immunostained for neurofilament (NF, axon marker), neural cell adhesion molecule (NCAM, non-myelinating Schwann cell marker), and myelin basic protein (MBP, myelinating Schwann cell marker) (**Fig. 3c–d,** control nerve **Fig. 3 e–f**) [4]. Consistent with staining from a prior biopsy [1], the staining showed a severe loss of large (white arrow) greater than small (purple arrow), myelinated axons and a moderate loss of small, non-myelinated axons. The dorsal root ganglia showed frequent Nageotte nodules, indicating ganglion cell loss (**Fig. 3b**). The ventral roots were unremarkable.

## Discussion

In comparison to ACOX LOF, Mitchell syndrome has notable differences in the gross presentation and distribution of pathology. LOF is associated with prominent brain involvement [5], while GOF has early and persistent burden of disease in the spinal cord that ultimately involves the brain. However, ACOX1 LOF has limited reports evaluating the peripheral nervous system components; therefore, it cannot be ruled out that both conditions affect the peripheral nervous system and spinal cord. In LOF the cerebrum and cerebellum show prominent atrophy [5], while GOF results in a grossly normal brain. In LOF, the cerebral, cerebellar, and brainstem white matter were severely affected; these regions were less affected in GOF. Both conditions have macrophage infiltration in affected areas. Under electron microscopy, macrophages in LOF samples show spiculated inclusions in membrane-bound aggregates [5]. However, these aggregates were not seen on peripheral nerve electron microscopy in the GOF case [1].

Differences in disease distribution between these conditions suggest selective vulnerability of specific cell types. For example, peripheral axonal loss with dorsal root neuronal dropout and destruction of the dorsal columns suggest selective vulnerability of primary sensory afferent neurons. Afferent sensory neuron vulnerability is supported by the relative preservation of the ventral root and the presenting symptom of sensory ataxia. Preferential early involvement for afferent neurons/axons could also underlie the early hearing loss in these patients and both peripheral and central nervous system involvement. To better understand this selective vulnerability of the primary sensory afferent neurons, animal models with dorsal root ganglia may be beneficial. However, sensory neurons are not the only cell type affected, as demonstrated by the early T2-hypertensity and obvious loss of myelin. In addition, the neuron loss in the ventral horn and corticospinal tract degeneration indicates eventual motor neuron vulnerability, findings compatible with the later development, clinically, of quadriparesis and neuromuscular respiratory failure.

While both gain- and loss-of-function of ACOX1 affect myelin and neurons, Mitchell syndrome provides stronger support for a neuronal contribution to the pathology rather than ACOX1 LOF. The findings of axon degeneration in the peripheral nervous system on electron microscopy [1], without a preponderance of naked axons, suggest that this is not a myelin-driven process early in disease in the peripheral nervous system. However, based on the distinct T2-hyperintensity in the dorsal column, myelin involvement cannot be ruled out. The areas of pallor identified in this autopsy show relatively proportional disruptions to myelin and axons based on LFB-PAS and NF respectively, making it difficult to infer chronology of the injury. Therefore, both neurons and myelinating cells likely contribute to disease progression.

Several overexpression ACOX1 Mitchell Syndrome non-human models exist that show different phenotypes and do not fully align in their proposed mechanisms. These phenotypes partially align with what we report here in the human pathology. Studies overexpressing drosophila GOF ACOX1 in drosophila and human ACOX1 in rat Schwann cells highlight increased hydrogen peroxide, reactive oxygen species, and ensheathing glial cell death as a likely mechanism [1]. While overexpression of human ACOX1 in zebrafish showed no difference in oligodendrocyte precursor cell number, nor were differences detected in catalase or nitric oxide synthase 2A (nos2a) (markers of oxidative stress), it instead showed increased Activating Transcription Factor 4 (ATF4) expression, decreased peroxisomes, and decreased swimming [3]. Further studies are needed to evaluate the neuronal contribution to the zebrafish phenotype. A consistent feature among these distinct models with differing phenotypes is the rescue with N-acetylcysteine (NAC) amide or dendrimer-NAC, which can function as an antioxidant and cytoprotectant [1,3]. NAC amide allows for better blood brain barrier penetration but is limited to non-human models. Instead, NAC is available for human use and has been used clinically for numerous conditions, including Mitchell Syndrome. NAC may have partially improved symptoms, but has been insufficient to prevent disease progression, as evident in this patient [1]. While NAC/NAC amide are pervasive in the literature, the mechanisms of action are not agreed upon [6]. Other currently used treatments focus on neuroprotection and immunomodulation/immunosuppression [1]. There is no unifying model that reproduces the phenotypes seen in humans.

While ACOX1 loss- and gain-of-function are rare diseases, their pathology presents a unique opportunity to understand the contribution of critical metabolic pathways to the nervous system. The differences and similarities between these conditions highlight cell-specific vulnerabilities that can help us develop more accurate disease models and possibly provide clues to disease mechanisms.

## Conflict of interest statement

Robert Bucelli is a medical advisor for the Mitchell and Friends Foundation and is the principal investigator on the Mitchell and Friends Foundation Gift Fund.
